# Radiation Exposure During Lumbar Interbody Fusion Surgery Can Be Reduced by Using a Three-Dimensional Patient-Specific Template Guide

**DOI:** 10.7759/cureus.58787

**Published:** 2024-04-22

**Authors:** Yuki Nagashima, Tetsuhiro Ishikawa, Joe Katsuragi, Yasuhito Sasaki, Masahiro Suzuki, Tomotaka Umimura, Ryohei Sawada, Daisuke Hashiba, Makoto Yamamoto, Seiji Ohtori

**Affiliations:** 1 Orthopedic Surgery, Sanmu Medical Center, Sanmu, JPN; 2 Radiology, Sanmu Medical Center, Sanmu, JPN; 3 Orthopedic Surgery, Chiba University Hospital, Chiba, JPN; 4 Orthopedic Surgery, Graduate School of Medicine, Chiba University, Chiba, JPN

**Keywords:** transforaminal lumbar interbody fusion (tlif), posterior lumbar interbody fusion (plif), lumbar spinal canal stenosis, radiation exposure, fluoroscopy, patient-specific 3d template guide, cortical bone trajectory

## Abstract

Background: The use of posterior lumber interbody fusion (PLIF) using cortical bone trajectory (CBT) with a patient-specific 3D template guide is increasingly widespread. To our knowledge, no studies have extensively evaluated the reduction of radiation exposure when using patient-specific drill template guides. The purpose of this study is to compare the intra-operative radiation dose and surgeon’s exposure to radiation in CBT-PLIF when using a patient-specific drill guide with that in traditional minimally invasive (MIS)-PLIF.

Methods: In this observational study, we retrospectively compared data from five patients who were treated with single-level CBT-PLIF using a patient-specific drill guide (G group) and five patients who were treated with single-level traditional MIS-PLIF (M group). We compared the surgical time, surgeon’s exposure to radiation, and intra-operative radiation time and dose between the two groups of patients.

Results: The mean age of the patients was 67.0 years in the M group and 74.2 years in the G group. The average surgical time was 242.8 min in the M group and 189.6 min in the G group (p = 0.020). The surgeon’s exposure to radiation was 373.7 µSv in the M group and 81.75 µSv in the G group at chest level outside the protector (p = 0.00092); 42.0 µSv (M group) and 3.6 µSv (G group) at chest level inside the protector (p = 0.0000062); and 4.33 µSv (M group) and 1.20 µSv (G group) at the buttocks of the surgeon (p = 0.0013). Radiation time was 269.8 s (M group) and 56.6 s (G group) (p = 0.0097), and radiation dose was 153.7 mGy (M group) and 30.42 mGy (G group) (p = 0.00057).

Conclusion: The patient-specific drill template guide is an invaluable tool that facilitates the safe insertion of CBT screws with a low radiation dose from the outset.

## Introduction

A cortical bone trajectory (CBT) is a new technique for inserting pedicle screws that was disclosed in 2008 by Santoni et al. [1]. This new trajectory begins at the pars interarticularis and passes through the pedicle in a craniolateral direction, which reduces muscle dissection and incision length and increases the contact between the screw and cortical bone in both the vertebral body and pedicle [1-4]. These CBT characteristics indicate that this method is a desirable choice in terms of fixation strength and minimally invasive surgery. Furthermore, Matsukawa et al. reported many biomechanical and clinical studies of modified “long CBT” [2,5-8], which achieved both effective load transmission and maximum cortical purchase. These reports indicated the appropriate insertion point and ideal trajectory of long CBT. Over the past decade, as a substitute for the conventional pedicle screw approach, this method is becoming more popular. However, achieving an ideal trajectory for long CBT is challenging because of its narrow cortical corridor with fewer anatomical landmarks within a limited operative field [4,7,8]. Because of the intra-operative fluoroscopic guidance required to achieve an ideal trajectory to improve accuracy, there is a long learning curve and high exposure to radiation [7]. By contrast, a patient-specific three-dimensional (3D) template guide for pedicle screws has more recently been reported as a potential means of enhancing precision and safety [9,10]. A randomized cadaveric investigation of patient-specific guided pedicle screw placement showed that it was quicker and more accurate than inserting pedicle screws by hand under fluoroscopy [11]. Matsukawa et al. reported that CBT using a patient-specific template guide reduced intraoperative radiation time compared with freehand CBT [12]. On the other hand, minimally invasive posterior lumber interbody fusion (PLIF (MIS-PLIF) is widely performed because of its versatility, and although it requires the use of fluoroscopy, radiation exposure has been gradually reduced with advances in the technique [13]. Striano et al. identified that the average radiation exposure per case was 60.5 mGy for transforaminal lumbar interbody fusion (TLIF)/PLIF and that lateral approaches increased BMI, minimally invasive techniques, and more caudal operative levels as significantly associated with increased radiation exposure [13].

Using a patient-specific drill guide to insert the CBT screws into the optimal insertion trajectory may reduce the surgeon’s exposure to radiation and radiation dose during surgery. Furthermore, to our knowledge, no studies have extensively evaluated the reduction in the intra-operative radiation dose and surgeon’s exposure to radiation when using patient-specific drill template guides in clinical practice. Therefore, we sought to compare the intra-operative radiation dose and surgeon’s exposure to radiation in CBT-PLIF when using a patient-specific drill guide with that in traditional MIS-PLIF.

## Materials and methods

In this observational study, we retrospectively compared data from five patients treated with single-level CBT-PLIF using a patient-specific drill guide (G group) and five patients treated with single-level, traditional MIS-PLIF (M group) from November 2020 to December 2021. The following conditions have to be met for the interbody fusion to be considered: degenerative spondylolisthesis, single-level, low-grade (Meyerding grade I or II), and severe back and leg pain that was not relieved by conservative treatments. All the surgeries were performed by the same surgeon in both groups. It takes at least two weeks from planning the guide until it is delivered to the hospital. Therefore, CBT-PLIF was performed in cases where the guide could be delivered before surgery, and MIS-PLIF was performed in all other cases. Data from patients with multilevel fusion surgery and reoperations affecting intra-operative radiation time were excluded.

In the M group, every single screw had been inserted transfascially with a percutaneous pedicle screw (PPS) trajectory in all cases. First, the skin was incised medially, and bilateral facetectomy was performed for decompression, and the surgeon used one-shot fluoroscopic confirmation of lateral images to place two interbody cages with an autograft as anteriorly as possible. Then, the fascia was expanded laterally over the fascia and was incised at the screw insertion position. The screws and rods were inserted using a PPS system under fluoroscopic guidance, and the surgeon confirm one-shot fluoroscopy at least once during each procedure.

**Figure 1:**
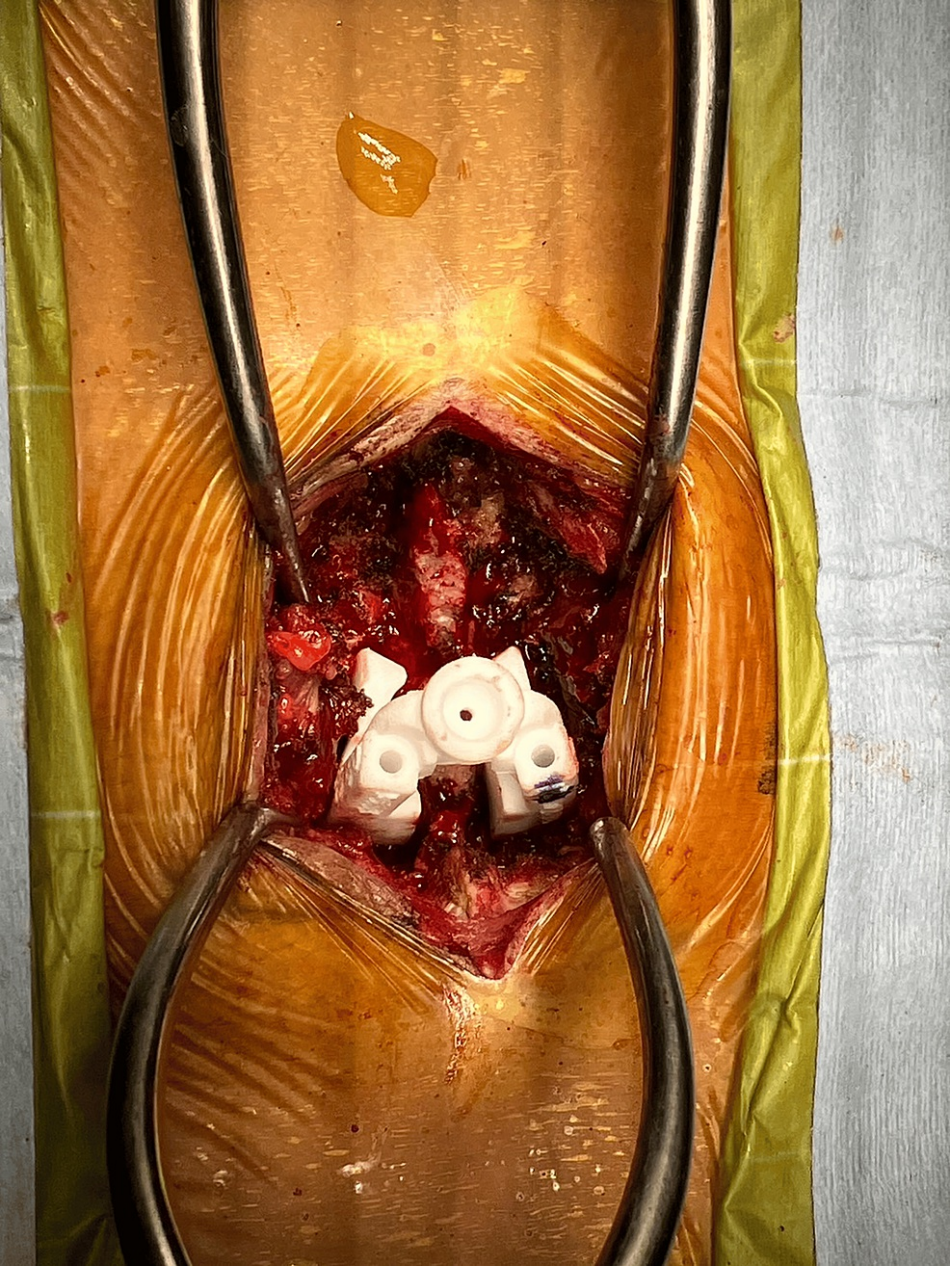
Intra-operative photograph: the 3D guide was placed on the bone surface

In the G group, surgery was performed using MySpine (Medacta International, Castel San Pietro, Switzerland) as a patient-specific 3D template guide. After computed tomography (CT) of lumbar vertebral bones had been acquired with a slice thickness of 0.5-1.0 mm, a 3D model of each vertebra was reconstructed using the program for processing 3D medical images (Mimics; Materialise, Leuven, Belgium). Then, a surgical plan for screw placement, including screw position at the center of the pedicle, screw length, screw diameter, and screw direction in sagittal/transverse trajectory angles, was developed by the surgeon using 3D CAD design software (Solidworks; Dassault Sysètmes, Vélizy-Villacoublay, France). According to a previous study, the planned trajectory for long CBT was set as described by Matsukawa et al. [7]. A 3D CBT screw placement guide was made based on the planned screw trajectory for each vertebra. A midline incision was made, and the paraspinal muscles were dissected to expose the lateral margins of the pars interarticularis. After exposure of the posterior bone surface, including the caudal end of the lamina and central part of the lamina, which were the main contact areas of the 3D guide, the guide was placed on the bone surface (Figure 1).

The guide was installed before decompression surgery, and if the lower facet joints overlapped the entry points of the screws, the tips of the lower facet were removed. The surgeon performed one-shot fluoroscopic confirmation of lateral images at least three times each before drill insertion, after drill insertion, and after temporary insertion pin placement. Decompression, interbody treatments, bone grafting, and screw insertion were carried out after the screw path was created. Bilateral facetectomy was performed for decompression in this series, and the surgeon used one-shot fluoroscopic confirmation of lateral images to place two interbody cages with an autograft as anteriorly as possible the same as MIS-PLIF. To prevent inadvertent cortical fissures during screw insertion, the screw pathways were tapped to the same size as the intended screws. Finally, screws with preoperatively planned lengths and diameters were inserted, and rods were connected.

We compared the surgical time, radiation time and dose, and the surgeon’s exposure to radiation between the two groups. All radiation time and dose were determined using a C-arm fluoroscopic system (Oec One, GE Health Care, IL), which can measure radiation time (s: second) and dose (milligray: mGy). The surgeon’s exposure to radiation was measured at three sites: chest level outside the protector, chest level inside the protector, and buttocks of the surgeon using real-time radiation dosimeters (Mydose mini; Hitachi Healthcare Manufacturing, Chiba, Japan). The dosimeter is for medical use and for personal measurement and can accurately detect exposure ranging from 1 to 1,000 millisieverts (mSv), and the surgeon’s exposure dose to radiation was recorded in microsieverts (μSv).

In the G group, the superimposed preoperative and postoperative reconstructed vertebra were then examined for any screw deviations from the planned parameters (vertical deviation at the entry point, horizontal deviation at the entry point, insertion direction of sagittal angle, and insertion direction of transverse angle), which were measured using 3D CAD design software. We could not make similar comparisons in group M because we did not use planning with 3D models.

This study is a retrospective case series of 10 patients with lumbar spinal canal stenosis patients who underwent posterior decompression and fixation surgery. This study was authorized by the institutional ethics committee, and informed consent was obtained from all participants. A Student t-test was used to compare each parameter, and differences with p < 0.05 were considered significant.

## Results

The mean age of the patients was 67.0 (range: 51-80) years in the M group and 74.2 (range: 70-82) in the G group. There were two women and three men in the M group and one woman and four men in the G group. The average BMI was 26.3 (range: 22.15-29.90) in the M group and 24.70 (range: 17.78-29.41) in the G group; there was no significant difference between the two groups (p = 0.26) (Table 1).

**Table 1:** Characteristics of patients Group M: patients with single-level traditional MIS-PLIF, Group G: patients with single-level CBT-PLIF using a patient-specific drill guide, F: female, M: male, BMI: body mass index

No.	Group	Age (y)	Sex	Level	Height (cm)	Body weight (kg)	BMI
1	M	51	M	L3/4	170	83	28.7
2		59	M	L5/S	170	64	22.1
3		79	F	L4/5	155	62	25.8
4		66	M	L5/6	167	83.4	29.9
5		80	F	L4/5	150	56	24.9
6	G	82	M	L4/5	160	61.5	24.0
7		70	M	L1/2	168	83	29.4
8		76	M	L5/S	160	63	24.6
9		72	M	L4/5	164	74.4	27.7
10		71	F	L4/5	150	40	17.8

The blood loss, surgical time, intra-operative radiation time, radiation dose, and surgeon’s exposure to radiation are shown in Table 2.

**Table 2:** Results of blood loss, surgical time, radiation time, radiation dose, and surgeon’s radiation

Patient no.	Group	Blood loss (mL)	Surgical time (min)	Radiation exposure time (s)	Radiation dose (mGy)	Radiation exposure (outside/inside/buttocks, mSv)
1	M	50	222	449.12	233.2	359/48/2
2		150	228	246.07	117	384/52/7
3		80	221	336.57	181.5	363/32/3
4		700	238	147.99	120.9	440/42/5
5		100	305	169	115.95	322/36/6
6	G	200	164	65.28	33.2	67/2/6
7		10	157	38.25	29.5	89/5/0
8		120	185	94	61.03	165/5/0
9		200	240	36.07	20.64	83/6/0
10		50	202	49.3	7.72	6/0/0

**Figure 2:**
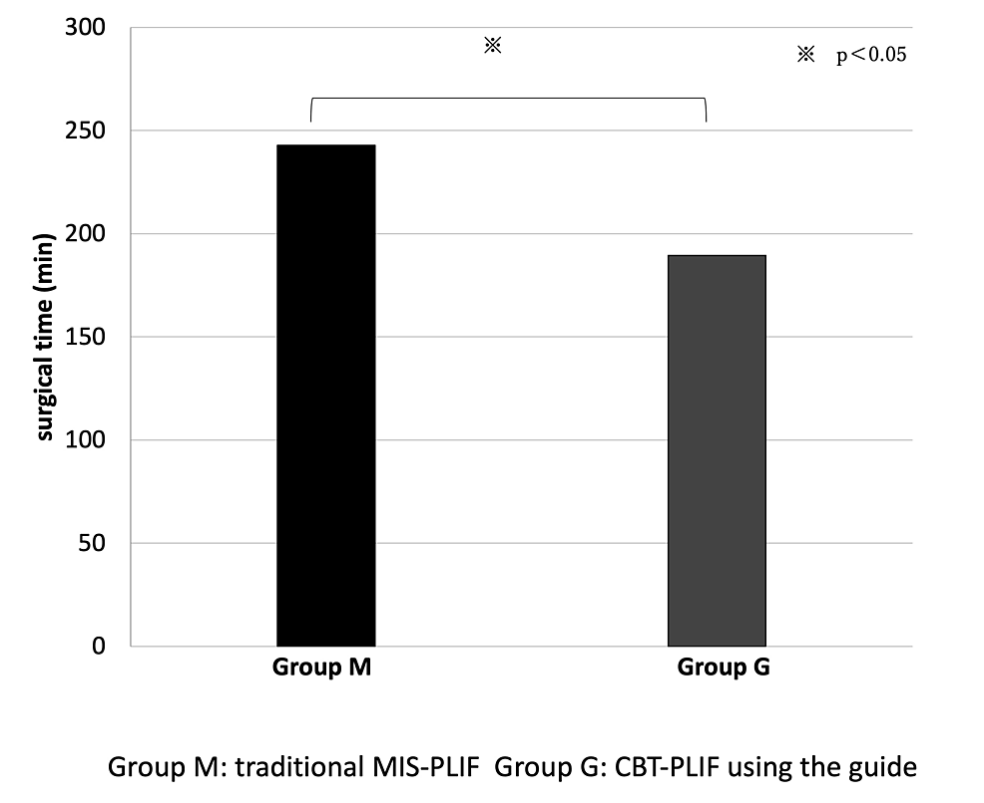
The average surgical time

The average surgical time in the G group (189.6 min) was significantly shorter than that in the M group (242.8 min) (p = 0.020) (Figure 2).

**Figure 3:**
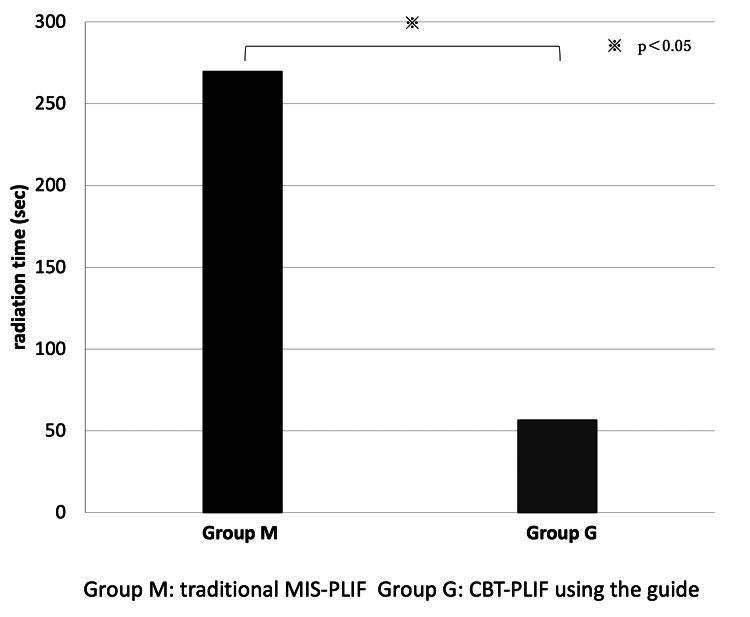
The average radiation time

The average radiation time in the M group (269.8 s) was significantly longer than that in the G group (56.6 s) (p = 0.0097) (Figure 3).

**Figure 4:**
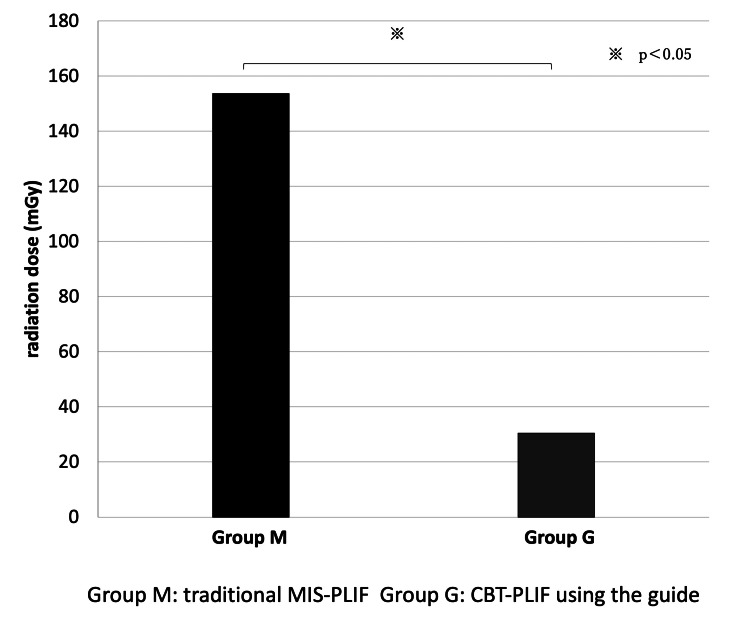
The average radiation dose

The average radiation dose in the M group (153.7 mGy) was significantly higher than that in the G group (30.42 mGy) (p = 0.00057) (Figure 4).

**Figure 5:**
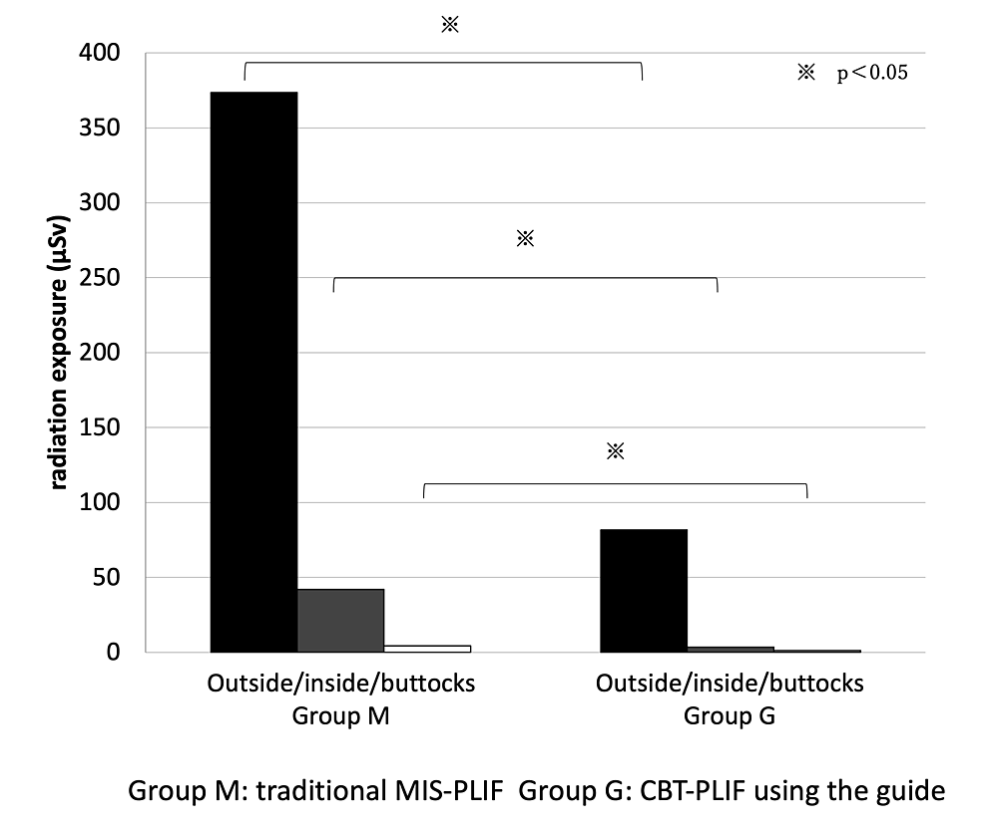
The surgeon’s average exposure to radiation

The surgeon’s average exposure to radiation was significantly higher in the M group (373.7 mSv) than in the G group (81.75 mSv) at chest level outside the protector (p = 0.00092); in the M group (42.0 mSv) than in the G group (3.6 mSv) at chest level inside the protector (p = 0.0000062); and in the M group (4.33 mSv) than in the G group (1.20 mSv) at the buttocks of the surgeon (p = 0.0013) (Figure 5).

No patients required screw replacement intra-operatively, and postoperative CT showed no screw deviation in any patient in both groups. In G group, at the entry point of the inserted screws, the average vertical deviation from the planned parameters was 1.16 (range: 0.05-3.46) mm, the horizontal deviation was 1.02 (range: 0.21-2.29) mm, the deviation of the insertion direction of sagittal angle was 3.17° (range: 0.49°-5.21°), and the transverse angle was 4.08° (range: 1.18°-10.94°) (Table 3).

**Table 3:** Deviations of the inserted screws from the planned parameters in the G group "Superior" refers to the screw at the cranial side of the fixed level, and "Inferior" refers to the screw at the caudal side of the fixed level. "Left" refers to the screw on the left side, and "right" refers to the screw on the right side.

Patient no.	Δ Vertical deviation (mm)				Δ Horizontal deviation (mm)				Δ Sagittal angle (°)				Δ Transversal angle (°)			
	Superior		Inferior		Superior		Inferior		Superior		Inferior		Superior		Inferior	
	Left	Right	Left	Right	Left	Right	Left	Right	Left	Right	Left	Right	Left	Right	Left	Right
6	0.05	1.32	1.24	2.78	0.80	1.37	0.50	1.78	2.28	0.69	0.34	2.47	7.49	2.59	1.13	3.48
7	3.46	1.45	0.30	0.21	0.60	0.45	0.75	1.15	1.15	0.06	3.23	2.08	1.06	1.15	2.23	2.72
8	0.10	0.01	2.04	1.73	0.32	2.01	2.29	1.05	0.31	0.83	8.85	5.98	4.33	0.16	9.13	3.32
9	1.03	1.72	1.33	0.27	0.66	1.6	1.08	1.19	0.15	2.50	0.41	2.83	1.92	2.10	0.92	2.91
10	0.41	0.49	1.35	1.98	0.42	0.21	1.84	0.30	0.01	1.68	2.99	2.97	3.54	1.96	1.08	11.66

No patients required screw replacement intra-operatively, and postoperative CT showed no screw deviation in any patient.

## Discussion

The present study sought to investigate the intra-operative radiation dose and surgeon’s exposure to radiation during the surgery and the accuracy and safety of CBT screw placement using a patient-specific template guide. The results showed that using a patient-specific template guide reduced the surgical times, radiation dose, and the surgeon’s exposure to radiation. Moreover, all the screws were safely placed in accurate positions in the G group without any perforation.

In the present study, the surgical time was significantly shorter in the G group than it was in the M group. Less invasiveness is considered one of the advantages of CBT [1,2]. CBT trajectory follows a laterally directed path in the transverse plane as the starting points for the screw are more medial than they are in conventional trajectories [1-4]. Thus, CBT screw insertion does not require excessive dissection, which is advantageous for reduced preservation of the posterolateral branch of the lumbar nerve root and muscle dissection [2]. In these respects, CBT-PLIF allows screw insertion without greater invasiveness and may have reduced operative time compared with MIS-TLIF. Heyde et al. reported that patient-specific guides could reduce operative time [14]. In the present study, only single-level fusion surgery was included, and patients requiring reoperations were excluded, suggesting that using patient-specific guides may have reduced operative time. The disadvantage of using a drill guide for a conventional pedicle screw trajectory is the less extensive muscle dissection. From this perspective, CBT and patient-specific guides are a compatible combination.

In the present study, the radiation time, dose, and the surgeon’s exposure to radiation were significantly lower in the G group than they were in the M group. Maruo et al. reported that the patient-specific 3D template guide reduced operating time, but did not describe radiation doses in detail [15]. Yamashita et al. noted that a higher exposure at the lateral lumbar image is due to the thickness of the soft tissues of the trunk [16]. However, as there was no significant difference in BMI between the two groups in the present study, the difference in radiation exposure might be due to differences in surgical technique. The use of the guide eliminates the need for AP fluoroscopy, which is helpful because of the complexity of the surgical procedure and radiation exposure during surgeries. Hyun et al. reported that robotic surgery allows safe screw insertion with less radiation exposure [17], but the high cost of implementation is a problem. The present technique benefits both the patients and the surgeons because it achieves a low radiation exposure, radiation time, and radiation dose. Another advantage is that patient-adapted drill guides can be introduced at any facility at a relatively low cost.

All the screws were safely placed in accurate positions without any perforation in the G group. Kasukawa et al. reported a deviation rate of 10% in CBT-TLIF using fluoroscopy, which was better than TLIF with conventional PPS [4]. Several authors have reported the accurate placement of CBT screws using a patient-matched, 3D-printed guide in lumbar spinal surgery [9-11,14,15]. Lu et al. reported that conventional pedicle screws could be accurately inserted in scoliosis surgery using a 3D-printed navigation template [18], Matsukawa et al. reported that sacral-arar-iliac screws could be accurately inserted using a 3D patient-specific template guide [19], and Hirao et al. reported that cervical pedicle screws are also inserted using this patient's specific templating system [20]. The findings of this study indicate that using a patient-specific drill template guide facilitates precise screw placement with reduced radiation exposure.

The present study has several limitations. First, the sample size is small (five cases each), and the data for the patient-specific drill template guide are derived from its initial introduction. We consider this paper as a preliminary study, estimating the approximate radiation exposure of CBT-PLIF using a patient-specific drill guide. Second, the control is traditional MIS-PLIF using PPS under fluoroscopic guidance and not a direct comparison of CBT-PLIF using fluoroscopic guidance and CBT-PLIF using a patient-specific drill template guide. However, because achieving an ideal trajectory for long CBT by hand is challenging and requires frequent fluoroscopy, we considered that MIS-PLIF requires less fluoroscopy than manual CBT. Third, the impact of direct and indirect radiation exposure was not investigated.

## Conclusions

In conclusion, the intra-operative radiation time, dose, and surgeon’s exposure to radiation were significantly lower for the group of patients who underwent CBT-PLIF using a patient-specific drill template guide than for the group of patients who underwent traditional MIS-PLIF. All the screws were safely placed in accurate positions without any perforation using a patient-specific drill template guide. A patient-specific drill template guide is an invaluable tool that facilitates the safe insertion of CBT screws with a low radiation dose from the outset.
